# Oxidative status in plasma, urine and saliva of girls with anorexia nervosa and healthy controls: a cross-sectional study

**DOI:** 10.1186/s40337-021-00408-6

**Published:** 2021-04-21

**Authors:** Alexandra Gaál Kovalčíková, Ľubica Tichá, Katarína Šebeková, Peter Celec, Alžbeta Čagalová, Fatma Sogutlu, Ľudmila Podracká

**Affiliations:** 1 Department of Paediatrics, The National Institute of Children’s Diseases and Faculty of Medicine, Comenius University, Limbová 1, 83340 Bratislava, Slovakia; 2 Institute of Molecular Biomedicine, Faculty of Medicine, Comenius University, Bratislava, Slovakia; 3 Institute of Pathophysiology, Faculty of Medicine, Comenius University, Bratislava, Slovakia; 4 Department of Molecular Biology, Faculty of Natural Sciences, Comenius University, Bratislava, Slovakia; 5 Department of Medical Biology, Faculty of Medicine, Ege University, Bornova, Izmir, Turkey

**Keywords:** Eating disorder, Malnutrition, Oxidative status, Antioxidants, Biomarkers

## Abstract

**Background:**

Anorexia nervosa (AN) is a serious psychosomatic disorder with unclear pathomechanisms. Metabolic dysregulation is associated with disruption of redox homeostasis that might play a pivotal role in the development of AN. The aim of our study was to assess oxidative status and carbonyl stress in plasma, urine and saliva of patients with AN and healthy controls.

**Methods:**

Plasma, spot urine, and saliva were collected from 111 girls with AN (aged from 10 to 18 years) and from 29 age-matched controls. Markers of oxidative stress and antioxidant status were measured using spectrophotometric and fluorometric methods.

**Results:**

Plasma advanced oxidation protein products (AOPP) and advanced glycation end products (AGEs) were significantly higher in patients with AN than in healthy controls (by 96, and 82%, respectively). Accordingly, urinary concentrations of AOPP and fructosamines and salivary concentrations of AGEs were higher in girls with AN compared with controls (by 250, and 41% in urine; by 92% in saliva, respectively). Concentrations of thiobarbituric acid reactive substances (TBARS) in saliva were 3-times higher in the patients with AN than in the controls. Overall antioxidants were lower in plasma of girls with AN compared to the controls, as shown by total antioxidant capacity and ratio of reduced and oxidized glutathione (by 43, and 31%, respectively).

**Conclusions:**

This is the first study assessing wide range of markers of oxidative status in plasma, urine and saliva of the patients with AN. We showed that both, higher levels of markers of oxidative stress and lower antioxidants play a role in redox disruption. Restoration of redox homeostasis might be of the clinical relevance

**Supplementary Information:**

The online version contains supplementary material available at 10.1186/s40337-021-00408-6.

## Plain English summary

Anorexia nervosa is a serious psychosomatic disorder with increasing incidence worldwide. Numerous general medical complications affecting most major organ systems are caused by progressive malnutrition and rapid weight loss. However, precise pathomechanism involved in the pathogenesis of anorexia nervosa remains to be elucidated. A potential role of oxidative stress caused by metabolic disruption in the etiology of anorexia nervosa was already described in plasma or blood components. This study aimed to investigate whether redox imbalance could be detected even in alternative body fluids of patients with anorexia nervosa - urine and saliva. Samples from female patients (*n* = 111) with anorexia nervosa and healthy controls (*n* = 29) were collected and analysed in this study. Our results indicated that changes in redox homeostasis could be detected not only in plasma, but also in urine and saliva of patients with anorexia nervosa. This was shown by higher markers of oxidative stress - advanced oxidation protein products, fructosamines and advanced glycation end-products. Oxidative damage of biomolecules might have direct cytotoxic effect associated with increased expression of proinflammatory cytokines, growth factors etc. Thus, many medical complications of anorexia nervosa might be directly connected to the accumulation of oxidatively damaged biomolecules.

## Introduction

Anorexia nervosa (AN) is a serious mental disorder characterized by an intense fear of weight gain and by a disturbed body image, which motivates severe dietary restriction or other weight loss behaviours (e.g. purging, excessive physical activity). Its prevalence among adolescents is increasing worldwide over the last few decades [1]. Metabolic dysregulation and homeostasis disruption that are directly attributable to extreme weight loss and malnutrition play an important role in dermatological, osteological [2], neuroendocrine [3], gastrointestinal [4], cardiovascular, and immune system disorders [5] leading to premature death [2], but precise pathomechanisms are not fully understood.

**Fig. 1:**
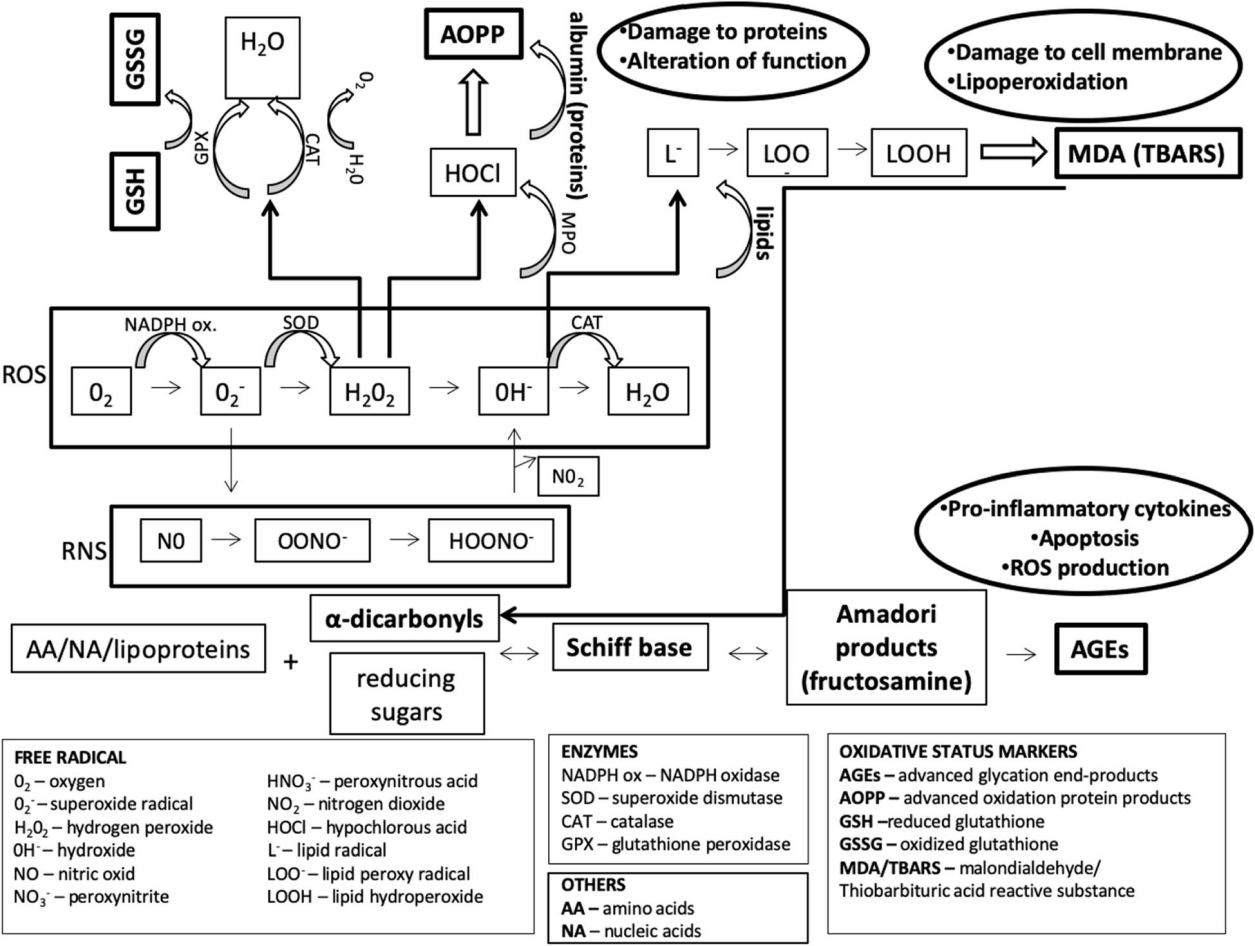
Mechanisms involved in oxidative stress. ROS – reactive oxygen species, RNS – reactive nitrogen species. ROS and RNS are particles with unpaired electrons. During redox homeostasis, increased production of ROS is balanced by their elimination by endogenous and exogenous antioxidants. An imbalance between production of prooxidants and antioxidants in favour of prooxidants results in oxidative stress

Oxidative stress is an imbalance between the production of free radicals (e.g., reactive oxygen species – ROS; reactive nitrogen species – RNS; Fig. 1) and the antioxidants caused either by the overproduction of the free radicals, the insufficiency of an antioxidant defence mechanism, or by a combination of both [6]. Oxidative stress may damage the biomolecules, impair the cell structures, and deteriorate the organ functions (Fig. 1) [7]. It activates various transcription factors that are involved in several pathological processes, including inflammation and immunity [8].

Since redox reactions are intracellular processes and a half-life of ROS is short, in clinical practice, oxidative status is usually quantified indirectly, via determination of concentrations of damaged substrates, often using non-specific methods. Malondialdehyde (MDA) is the end-product of lipid peroxidation. Thiobarbituric acid reactive substance (TBARS) assay is a standard non-specific method for the measurement of the levels of MDA and other aldehydes that might react with thiobarbituric acid [9]. Advanced oxidation protein products (AOPP) are formed by myeloperoxidase, during neutrophil activation [10]. The most common markers of carbonyl stress - fructosamines and advanced glycation end-products (AGEs) are formed during early and late stage, respectively, of non-enzymatic glycation of amino groups of proteins by the carbonyl compound of sugars or reactive aldehydes [11, 12]. A ratio of reduced (GSH) to oxidized glutathione (GSSG) serves as a marker of antioxidant status. GSH is considered to be one of the most essential scavengers of the free radicals [13]. Ferric reducing antioxidant power (FRAP) and total antioxidant capacity (TAC) evaluate overall endogenous and exogenous antioxidant capacity, without need for measuring of the individual antioxidants [14, 15].

Oxidative stress is associated with various mental disorders that might be interrelated to AN, including depression or anxiety [16]. Compared to the healthy controls, the patients with depression presented lower activities of the antioxidant enzymes, such as superoxide dismutase and catalase or lower concentrations of glutathione [17, 18]; and higher levels of MDA or AOPP [19, 20]. Moreover, the antidepressants and the psychotherapy increased the concentrations of the antioxidants and led to the decrease of MDA [19].

So far, only a few studies evaluated the oxidative status in patients with AN [21–23]. It has been shown that the effective treatment resulting in weight gain is associated with an improvement of oxidative status in patients with AN [23, 24]. Nevertheless, these studies mostly focused on plasma or blood components [21–23]. Saliva and urine represent a promising alternative to the blood sampling, as their collection is non-invasive, painless and does not require trained personnel [25].

Thus, the aim of our study was to compare the markers of oxidative status and carbonyl stress in different body fluids, including plasma, urine and saliva in the patients with AN and in the healthy controls. We hypothesised that reduced food intake and metabolic disruption lead to an increased production of oxidative - and carbonyl-stress markers and a decrease in the levels of antioxidants that would be manifested not only on the plasma level, but also in the alternative body fluids – urine and saliva.

## Methods

### Participants

In this study, 111 consecutive female patients aged from 10 to 18 (median age: 14.9, interquartile range - IQR: 13.4–16.1) years, hospitalized at the Paediatric endocrinology clinic or the Psychiatric clinic of the National Institute of Children’s Diseases (NICD) from February 2016 to February 2020 with a diagnosis of restrictive subtype of AN were enrolled. Diagnosis of AN was based on the new Diagnostic and Statistical Manual of Mental Disorders 5th Edition (DSM-5) [26]. E.g., the patient had to meet the following criteria: persistent restriction of energy intake that leads to a low body weight, intense fear of gaining weight, distorted perception of body weight and shape, undue influence of weight on self-evaluation or lack of the recognition of severity of the illness [26]. Manifestation of other psychiatric comorbidities (e.g., binge eating/purging subtype of AN, depression, anxiety) as diagnosed by the psychiatrists, and the presence of peripheral oedema (indicating volume overload) were the exclusion criteria. All anthropometric measurements and collections of samples in patients with AN were performed at the time of recruitment i. e. before initiation of refeeding, and pharmacological treatment.

Twenty-nine healthy age-matched girls (median age: 14.0; IQR: 11.0–16.3), recruited from regular check-ups in the practice of a general paediatrician at the same institution served as healthy controls. Inclusion criteria were BMI between 90th and 10th percentile for sex and age, no past or current mental disorder, and no involvement in weight reduction regimen [26].

Other exclusion criteria for all participants were pregnancy, lactation, hormonal contraception, and acute or chronic inflammation (C-reactive protein - CRP>10 mg/l).

### Anthropometric and blood pressure measurements

Anthropometric measurements were performed by the trained nurses according to the standard protocols of the NICD clinics. Briefly, the height was measured in standing position using stable stadiometer; body weight was measured by digital scales. BMI was calculated. Standard deviation (SD) of height, weight, and BMI was expressed using the current reference data of Slovak children and adolescents [27]. Three blood pressure measurements were taken on a dominant arm after 5 min resting in sitting position, using an automated device (Omron HBP-1100, Kyoto, Japan). The mean of the last two measurements was recorded. Blood pressure SD was calculated according to the guidelines for percentiles of blood pressure in normal-weight children and adolescents [28].

### Samples collection and handling

Biological material was collected in the morning hours, between 6.00 and 8.00 am. Blood was collected after overnight fasting, from median cubital vein, into K_3_EDTA, lithium-heparin, and SST™ ΙΙ Advance tubes (BD Vacutainer Plastic Tube, Becton Dickinson, Czech Republic). At the Department of Clinical Biochemistry of NICD, blood was centrifuged; plasma was subjected to immediate blood chemistry analyses and aliquots were stored at − 80 °C for measurements of markers of oxidative status and carbonyl stress. Spot urine was collected into 50 ml sterile Falcon tubes (Sarstedt, Numbrecht, Germany). To prevent saliva contamination, all participants were asked not to drink, eat or teeth brush optimally 60 min before sampling. Unstimulated saliva was collected according to the standard protocol [25], by spitting for 10 min into 15 ml sterile Falcon tubes (Sarstedt, Numbrecht, Germany). Urine and saliva were centrifuged at 1600 g for 10 min to remove cell debris. Supernatants were frozen until analyses.

### Laboratory measurements

Plasma glucose, albumin, total cholesterol, high-density lipoprotein cholesterol (HDL-C), triacylglycerols (TAG), bilirubin, aspartate aminotransferase (AST), alanine aminotransferase (ALT), creatinine, urea, uric acid, and high sensitive CRP were analysed using standard laboratory methods (Cobas c501, Roche Diagnostics, Mannheim, Germany). Measurement of cystatin C was performed using Biolis 24i Premium analyser (Tokyo Boeki Machinery, Tokyo, Japan). Blood count analyses were conducted using the Sysmex XN-1000™ Hematology Analyser (Sysmex Group’s, Kobe, Japan).

All methods used for determination of markers of oxidative status and carbonyl stress are described in detail in the Additional file 1. Briefly, TBARS [9], AGE-associated fluorescence [11], and GSH and GSSH [13] were determined fluorometrically; spectrophotometric methods were used to quantify fructosamines [12], AOPP [10], FRAP [14], and TAC [15].

Creatinine in urine was measured using Jaffé method [29], while urinary proteins were quantified using the pyrogallol-red method [30]. Plasma proteins were determined using a commercial kit (SERVA Electrophoresis GmbH, Heidelberg, Germany). Measurements of oxidative status markers and carbonyl stress were performed on a Synergy HT Multi-Mode Microplate Reader (BioTekInstruments, Inc., Winooski, VT, USA); and all chemicals were purchased from Sigma Aldrich (Steinheim, Germany).

### Assessment of kidney functions

Glomerular filtration rate (eGFR) was estimated using the cystatin C based Schwartz formula [31]. Urine protein to creatinine ratio (PCR) was calculated.

### Statistical analysis

Total number of participants required for the study was estimated according to G Power analysis using G*Power 3.1.9.4 software (Universität Kiel, Germany) from our preliminary results on TAC, to 72 individuals (noncentrality parameter δ = 3.31; critical t = 1.67; df = 70; actual power = 0.95). Similar results were obtained when data on AOPP (noncentrality parameter δ = 3.34; critical t = 1.66; df = 94; actual power = 0.95; total samples size = 94) or fructosamines (noncentrality parameter δ = 3.36; critical t = 1.67; df = 62; actual power = 0.95; total samples size = 64) were evaluated. GraphPad Prism software v. 6.01 was used for statistical analysis (GraphPad Software, San Diego, California, USA). The normality of the data distribution was tested using the D’Agostino-Pearson omnibus test. Outliers were detected using the Grubbs´ test. Patients and controls were compared employing two-sided Student’s t-test. To compare not normally distributed data, Mann-Whitney U test was used. Spearman correlation analysis and regression analysis was performed. Data are expressed as a mean ± SD or as a median and IQR. For auxological data, which are directly dependent on division into patients and controls, *p* < 0.05 was considered statistically significant. Due to multiple comparison in the assessment of oxidative and carbonyl stress markers, two groups were tested against a Bonferroni-adjusted (Bonf.) alpha level of 0.0071 (0.05/7), 0.001 (0.01/7), and 0.0001 (0.001/7) for plasma or saliva, and 0.0083 (0.05/6), 0.002 (0.01/6) and 0.0002 (0.001/6) for urine.

## Results

In our cohort, the median of AN duration was 10.9 months (IQR: 6.1–18.1). Fifteen girls (13.5%) were presented with primary amenorrhea; while 65 (58.6%) suffered from secondary amenorrhea, with a median of duration of 9.0 months (IQR: 4.6–15.3).

### Anthropometric and blood chemistry data

Auxological data of patients and controls are presented in Table 1. Two groups did not differ by age, height, and height SD (*p*>0.05). Body weight, body weight SD, BMI, and BMI SD significantly differed between patients with AN and controls (*p*<0.001). Concentrations of endogenous antioxidants including albumin, bilirubin, and uric acid were lower in the patients compared with the controls (albumin; bilirubin: *p*<0.001; uric acid: *p*<0.01); however, none of the patients with AN presented hypouricaemia, hypobilirubinaemia or hypoalbuminemia. Concentrations of markers of liver function AST and ALT in girls with AN were within reference range. Concentrations of urea and TAG (*p*<0.05) were significantly higher, while glycaemia was lower (*p*<0.01) in the girls with AN compared with the controls albeit within the reference range. Concentrations of urinary PCR were higher in the AN group compared with the controls (*p*<0.001). Neither cholesterol, CRP, nor eGFR differed significantly (*p*>0.05) between the groups (Table 1).

**Table 1:** Clinical and biochemical characteristics of patients with anorexia nervosa and healthy controls

	Anorexia nervosa	Healthy controls	*p*
Age (years)	14.9 (13.4–16.1)	14.0 (11.0–16.3)	NS
BMI (kg/m^2^)	15.1 (13.7–16.8)	19.8 (18.9–21.2)	<0.001
BMI SD	−1.8 (−2.4–-1.1)	0.03 (− 0.4–0.8)	<0.001
Height (cm)	164.5 (159.5–169.7)	162.0 (148.0–169.5)	NS
Height SD	0.2 ± 1.1	0.5 ± 1.0	NS
Body weight (kg)	41.5 ± 8.3	52.1 ± 10.1	<0.001
Body weight SD	−1.0 (−1.6–-0.1)	0.4 (−0.1–0.9)	<0.001
Systolic blood pressure (mm Hg)	112 ± 11	ND	
Systolic blood pressure SD	0.05 ± 0.96	ND	
Diastolic blood pressure (mm Hg)	71 ± 8	ND	
Diastolic blood pressure SD	0.47 ± 0.74	ND	
Glucose (mmol/l)	4.3 (4.0–4.6)	4.7 (4.2–4.9)	<0.01
Cholesterol (mmol/l)	4.56 ± 0.96	4.39 ± 0.71	NS
HDL-cholesterol (mmol/l)	1.72 ± 0.41	1.14 ± 0.22	<0.001
Triacylglycerols (mmol/l)	0.76 (0.61–1.12)	0.72 (0.53–0.84)	<0.05
Albumin (g/l)	46.7 (44.1–48.9)	49.9 (47.5–51.8)	<0.001
Total proteins (g/l)	69.9 (66.0–73.5)	70.5 (67.6–73.2)	NS
Uric acid (μmol/l)	218 (195–258)	264 (216–309)	<0.01
Bilirubin (μmol/l)	3.8 (2.7–4.8)	9.3 (6.0–13.7)	<0.001
AST (μkat/l)	0.39 (0.32–0.47)	ND	
ALT (μkat/l)	0.34 (0.25–0.52)	ND	
Urea (mmol/l)	4.6 (3.4–5.6)	3.7 (3.0–4.7)	<0.05
Creatinine (μmol/l)	66 ± 13	72 ± 15	<0.05
Cystatin C (mg/l)	0.70 (0.62–0.83)	0.79 (0.69–0.83)	NS
eGFR (ml/min/1.73m^2^)	99 (84–110)	89 (84–100)	NS
C-reactive protein (mg/l)	0.1 (0.1–0.4)	0.2 (0.1–0.5)	NS
Leukocytes (10^9^/l)	5.4 (4.4–6.2)	ND	
Urinary PCR (mg/mmol)	35.5 (22.2–48.8)	15.7 (12.7–24.0)	<0.001

*AST* aspartate aminotransferase, *ALT* alanine aminotransferase, *BMI* body mass index, *eGFR* estimated glomerular filtration rate, *HDL* high density lipoprotein, *ND* not determined, *NS* non-significant, *PCR* protein to creatinine ratio, *SD* standard deviation; Results are expressed as mean ± SD (normally distributed data) or as median (interquartile range), (skewed data)

### Markers of oxidative stress

In patients with AN, plasma and urinary TBARS concentrations were similar to those in the control group (plasma: p_Bonf._>0.0071; urine: p_Bonf._ > 0.0083, Table 2). In whole group of participants, Spearman analyses revealed positive correlations between plasma and urinary concentrations of TBARS (r = 0.50, p_Bonf._<0.0001). Salivary concentrations of TBARS were 3-fold higher in the AN group compared with the controls (p_Bonf._<0.0001, Table 2).

**Table 2:** Concentrations of markers of oxidative and carbonyl stress in patients with anorexia nervosa and healthy controls

	Plasma			Urine			Saliva		
**Markers**	**AN**	**CTRL**	**p**_**Bonf**_	**AN**	**CTRL**	**p**_**Bonf**_	**AN**	**CTRL**	**p**_**Bonf**_
TBARS	5.74 (3.47–10.82)	3.88 (3.15–6.48)	NS	1.60 (0.38–6.73)	1.16 (0.28–1.45)	NS	0.48 (0.22–0.90)	0.11 (0.08–0.13)	<0.0001
	*μmol/l*			*μmol/mmol creatinine*			*μmol/l*		
AOPP	0.84 (0.54–1.26)	0.33 (0.23–0.84)	<0.0001	48.4 (25.7–81.3)	12.0 (9.8–16.6)	<0.0002	8.20 (2.8–23.4)	24.5 (6.1–34.9)	NS
	*μmol/g proteins*			*μmol/mmol creatinine*			*μmol/l*		
AGEs	0.030 (0.020–0.040)	0.023 (0.018–0.024)	<0.0001	ND	ND		0.47 (0.28–0.82	0.31 (0.16–0.43	<0.0071
	*g/g proteins*			*g/mmol creatinine*			*g/l*		
FRUCTOS	1.85 (1.39–2.20)	1.36 (1.24–1.97)	NS	0.72 (0.52–1.03)	0.48 (0.37–0.54)	_._<0.0083	0.21 (0.14–0.39)	0.09 (0.04–0.30)	NS
	*mmol/l*			*mmol/mmol creatinine*			*mmol/l*		
FRAP	592 ± 129	600 ± 153	NS	1555 (613–5309)	552 (395–819)	<0.0002	131 (66–267)	143 (105–161)	NS
	*μmol/l*			*μmol/mmol creatinine*			*μmol/l*		
TAC	393 (235–674)	879 (502–1164)	<0.0001	1652 (699–4533)	573 (481–958)	<0.0002	567 (260–776)	393 (234–541)	NS
	*μmol/l*			*μmol/mmol creatinine*			*μmol/l*		
GSH/GSSG	5.46 (4.51–9.16)	8.99 (6.03–12.51)	<0.0001	0.81 (0.70–1.14)	1.32 (0.78–1.54)	NS	0.66 (0.38–1.42)	0.37 (0.30–0.43)	<0.001
	*ratio*			*ratio*			*ratio*		

*AGEs* advanced glycation end products, *AN* anorexia nervosa, *AOPP* advanced oxidation protein products, *FRAP* ferric reducing antioxidant power, *FRUCTOS* fructosamines, *GSH/GSSG* a ratio of reduced and oxidized glutathione, *NS* non-significant, *TAC* total antioxidant capacity, *TBARS* thiobarbituric acid reactive substance; Results are expressed as mean ± SD (normally distributed data) or as median (interquartile range), (skewed data)

**Fig. 2:**
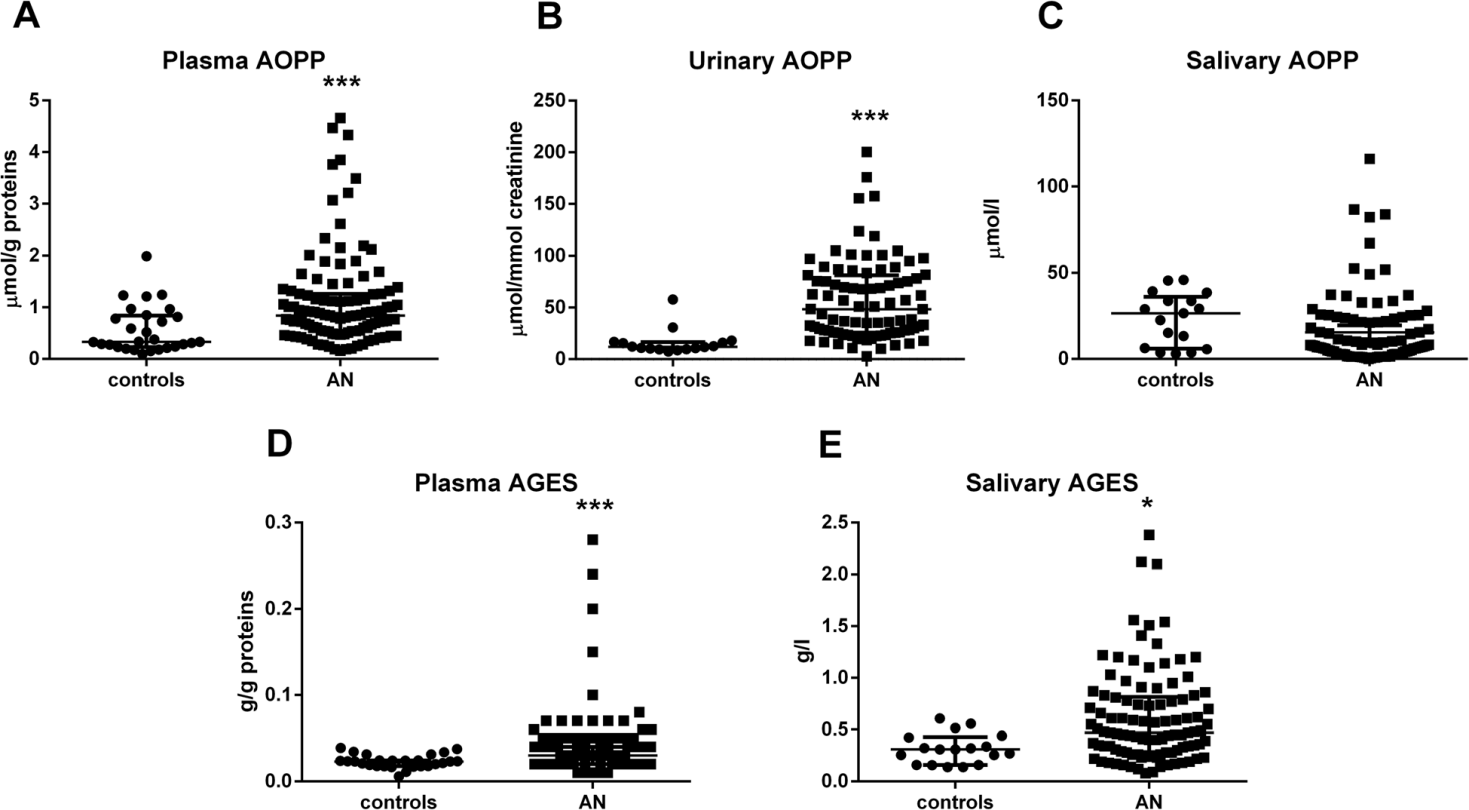
Concentrations of markers of oxidative and carbonyl stress. Concentrations of AOPP – advanced oxidation protein products in **a:** plasma, **b:** urine, **c:** saliva of the girls with AN and healthy controls. Concentrations of AGEs – advance glycation end products in **d**: plasma, **e**: saliva of the patients with AN and controls. Results are expressed as a median with interquartile range. * denotes Bonferroni adjusted *p*<0.0071 (for plasma and saliva), *** denotes Bonferroni adjusted *p*<0.0001 (for plasma and saliva) and *p*<0.0002 (for urine) in comparison to the control group (by Mann-Whitney test)

The group with AN displayed significantly higher concentrations of AOPP in plasma (by 96%, p_Bonf._<0.0001, Fig. 2a), as well as in urine (by 250%, p_Bonf._<0.0002, Fig. 2b) – compared with the controls. In the whole group of participants, Spearman analysis revealed a positive correlation between plasma and urinary AOPP (r = 0.48, p_Bonf._<0.0001). Salivary concentrations of AOPP in the girls with AN were similar to the controls (p_Bonf._ > 0.0071, Fig. 2c).

### Markers of carbonyl stress

In comparison to the healthy controls, the group with AN displayed significantly higher concentrations of AGE-Fl in plasma (by 82%, p_Bonf._<0.0001, Fig. 2d); as well as in saliva (by 92%, p_Bonf._<0.0071, Fig. 2e).

Concentrations of fructosamines in plasma, and saliva did not differed between girls with AN and controls (p_Bonf._ > 0.0071, Table 2), while urinary levels of fructosamines in girls with AN were higher by 41% - compared with controls (p_Bonf._<0.0083, Table 2).

### Markers of antioxidant status

Two groups did not differ in plasma, and salivary concentrations of FRAP (p_Bonf._>0.0071, Table 2). In comparison to controls, urinary concentrations of FRAP were 3-fold higher in girls with AN (p_Bonf._<0.0002, Table 2).

**Fig. 3:**
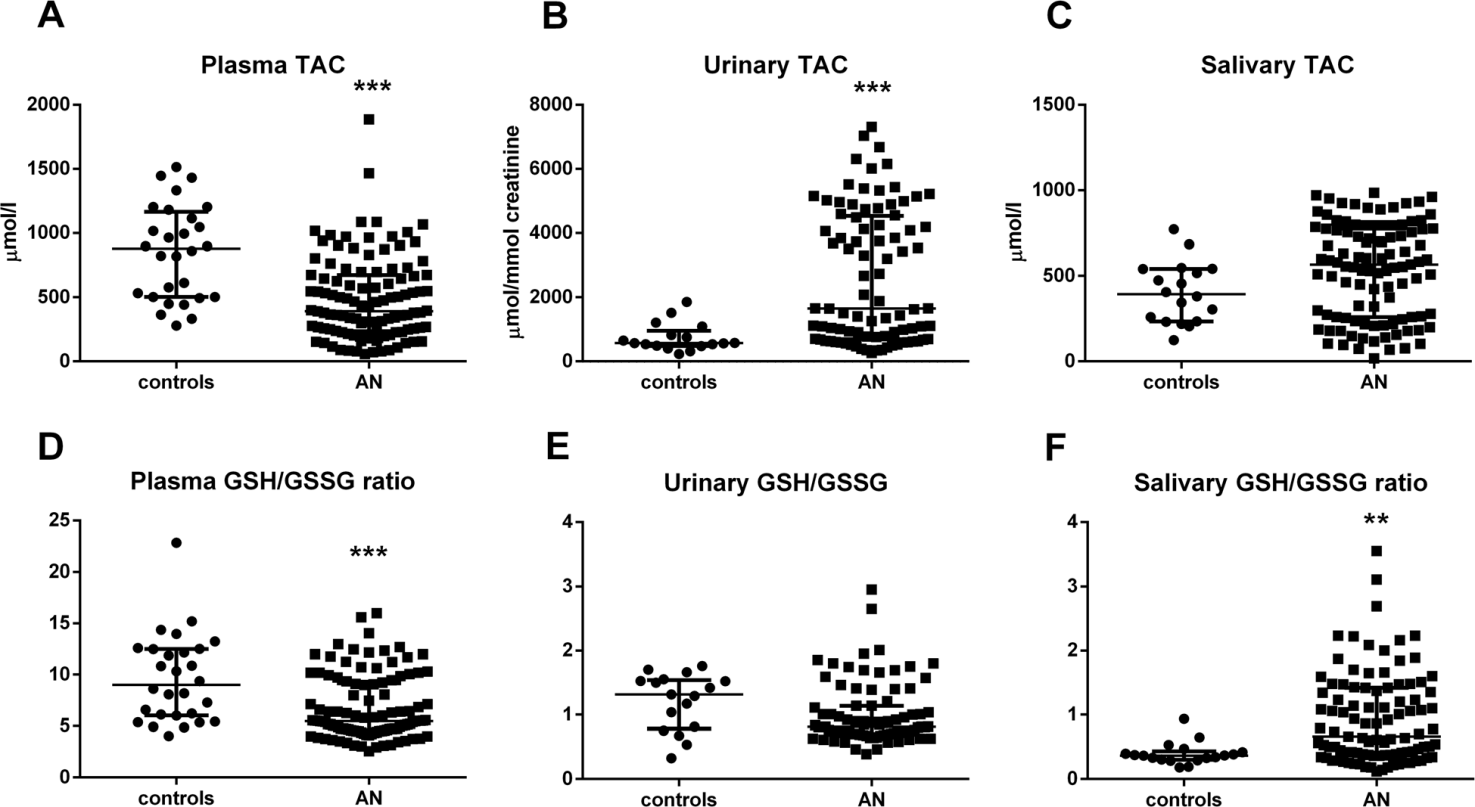
Concentrations of markers of antioxidant status. Concentrations of TAC – total antioxidant capacity in **a:** plasma, **b:** urine, **c:** saliva of the girls with AN and healthy controls. Concentrations of GSH/GSSG – a ratio of reduced/oxidized glutathione in **d:** plasma, **e:** urine, **f:** saliva of the patients with AN and controls. Results are expressed as a median with interquartile range. ** denotes Bonferroni adjusted *p*<0.001 (for plasma and saliva), *** denotes Bonferroni adjusted *p*<0.0001 (for plasma and saliva) and *p*<0.0002 (for urine) in comparison to the control group (by Mann-Whitney test)

Patients with AN had plasma TAC concentrations lower by 43% (p_Bonf._<0.0001, Fig. 3a), while urinary TAC concentrations were 3-fold higher - compared with controls (p_Bonf._<0.0002, Fig. 3b). Salivary TAC concentrations in patients with AN were similar to controls (p_Bonf._>0.0071, Fig. 3c).

In patients with AN, GSH/GSSG ratio in plasma was lower by 31% (p_Bonf._<0.0001, Fig. 3d), while in saliva it was higher by 132% (p_Bonf._<0.001, Fig. 3f) – compared to controls. In the whole group of participants, an inverse correlation between plasma and salivary GSH/GSSG was revealed using Spearman analysis (r = − 0.30, p_Bonf._<0.0071), as well as using regression analysis (beta = − 0.206, *p*<0.05). We did not find relation between plasma and salivary GSH (beta = 0.114, *p*>0.05), while negative relation between plasma GSSG and salivary GSSG was revealed (beta = − 0.304, *p*<0.001). Urinary of GSH/GSSG ratio did not differ between AN and control group (p_Bonf._ > 0.0083, Fig. 3e).

### The effect of the duration of AN

Regression analyses revealed positive relation between duration of the disease and plasma markers of oxidative stress – TBARS, AOPP (TBARS: beta = 0.294, *p*<0.01; AGEs: beta = 0.264, *p*<0.01) and carbonyl stress – AGEs (beta = 308, *p*<0.001). Moreover, a marker of antioxidant status TAC inversely correlated with disease duration (beta = − 0.250, *p*<0.001). While, positive relation was found between disease duration and urinary AOPP and TAC (AOPP: beta = 0.231, *p*<0.05; TAC: beta = 0.253, *p*<0.05, respectively), negative correlation was revealed with urinary fructosamines (beta = − 0.234, *p*<0.05; Additional file 2).

### The effect of BMI SD and menstrual status

In the whole group of participants, we found inverse correlations between BMI SD, and plasma AOPP (beta = − 0.143; *p*<0.05). Moreover, positive relation between BMI SD, and plasma TAC was revealed (beta = 0.213; *p*<0.05). Further, BMI SD correlated negatively with salivary TBARS and AGEs (TBARS: beta = − 0.200, *p*<0.05; AGEs: beta = − 0.234, *p*<0.01) and urinary AOPP, respectively (beta = − 0.227, *p*<0.05). Regarding the effect of menstrual status on oxidative status, inverse relation between the duration of secondary amenorrhea and FRAP was found (beta = − 0.369, *p*<0.01). Moreover, a trend to a positive correlation was revealed between LH and TAC (beta = 0.254, *p* = 0.06).

## Discussion

To the best of our knowledge, this is the first study assessing a wide range of markers of oxidative status and carbonyl stress in plasma, urine and saliva of the patients with AN. In addition to confirming former data that AN is associated with the markers of increased oxidative stress [22–24, 32], our data also suggest the presence of enhanced carbonyl stress. We confirmed our hypothesis that anorexia-associated redox imbalance and enhanced carbonyl stress are reflected by changes in their markers even in the alternative body fluids. However, the alterations in the markers assessed in non-invasively collected body fluids, i.e., urine and saliva, do not completely mirror those observed in plasma – considered as indicators of systemic changes.

Previous meta-analyses of Solmi et al. [23, 24] indicated a potential association between AN and systemic oxidative stress, as reflected by higher serum levels of oxidative stress marker apolipoprotein B and lower levels of antioxidants including superoxide dismutase, glutathione and albumin in patients with AN compared with the controls. Moreover, weight gain was associated with an improvement in oxidative status [23, 24].

Another studies demonstrated an impairment of oxidative status in blood elements [22, 32]. Erythrocyte tocopherol levels and superoxide dismutase activities were significantly lower in patients with AN compared to the controls [32]. Moreover, impairment of mitochondrial function associating with decreased mitochondrial O_2_ consumption, low GSH levels, and increased ROS production was revealed in leukocytes of the patients with AN [22].

ROS produced by mitochondria act as signalling molecules that are involved in different intracellular processes including autophagy. Autophagy is a catabolic process that is essential for the removal of cytoplasmic material or damaged organelles playing an important role in cellular homeostasis. During starvation or enhanced oxidative stress, autophagy is up-regulated to produce nutrients and to protect cells from apoptosis, respectively. An impairment of autophagy contributes to oxidative damage to lipids, proteins and nucleic acids especially due to slower mitochondria turnover. This bidirectional relation between oxidative stress and autophagy might be involved in various pathologies including AN [33].

Increased concentrations of plasma lipids in the patients with AN are associated with several mechanisms including alternation in hormonal regulation, and enhanced lipid reabsorption [34]. We assumed that the rise in plasma lipid levels in the patients with AN results in their higher susceptibility to oxidative damage and thus, systemic levels of TBARS would be elevated in patients with AN. Despite higher plasma lipid concentrations in girls with AN, plasma and urinary TBARS concentrations were not changed. On the other hand, higher salivary TBARS levels in the patients with AN could be connected to higher incidence of dental caries in patients with AN [35] that are implicated in higher salivary levels of TBARS [36]. Further, we observed inverse correlations of salivary TBARS and AGE-Fl with BMI. Previous study showed higher salivary MDA and AGEs in overweight and obese children compared with the normal weight children, and their positive correlation with BMI, respectively [37]. It seems that both obesity and underweight might affect salivary markers of oxidative stress.

Plasma AOPP are generated on proteins via hypochlorous acid produced by myeloperoxidase released from activated phagocytes [38], and may act as inflammatory mediators [39]. Thus, higher plasma AOPP levels in girls with AN compared with controls may indicate the activity of neutrophils or monocytes and inflammation. This is in line with previous study of Dalton et al. [40], showing that some inflammatory markers, including interleukin-6, interleukin-15 or tumor necrosis factor-beta are altered in patients with AN. However, the majority of quantified inflammatory markers including CRP, did not differ between patients with AN and healthy subjects [40]. Oxidative stress and changes in autophagy stimulate the release of proinflammatory cytokines [33]. In our study, CRP as a non-specific marker of inflammation did not differ between patients and controls. Increased plasma AOPP, however, suggest that the immune system is or at least was active. Given the lack of infection the inflammatory response can be judged as inadequate although more inflammatory markers are needed to better characterize the immune status [41] that might contribute to multiorgan dysfunction associated with AN. Since urinary oxidative status could reflect changes of local as well as systemic oxidative status [42], the rise of plasma concentrations of AOPP in girls with AN probably caused elevation of their urinary levels.

Fructosamines – reversible early glycation products reflect plasma glucose levels over approximately the last two weeks. However, in the presence of hydrogen peroxides, fructosamines might be formed on albumin even under normoglycaemic conditions, and may in turn contribute to protein damage via the generation of ROS [43]. In our study, we did not reveal any changes in plasma and salivary fructosamines. Similarly, to AOPP, the rise of urinary fructosamines could be attributed to higher protein content in urine of the girls with AN.

On the other hand, concentrations of AGEs – irreversible glycation products - were elevated in plasma and saliva of the girls with AN. Cytotoxic effects AGEs, mediated through ROS and inflammatory cytokines production, are implicated in various diseases [44]. Exogenous sources of AGEs, such as smoking or a high dietary intake of AGEs-rich foods [45], increased production under persistent hyperglycaemia [46], or decreased renal elimination [44] as sources contributing to the rise in plasma AGEs in AN might be excluded. Alternative pathways of AGEs formation in vivo include reactions of proteins with α-dicarbonyls, produced, among others, via lipid peroxidation [47]. However, we did not observe increased TBARS concentrations in patients with AN. On the other hand, previously shown local accumulation of peroxidated lipid products in liver of patient with AN [48] might indirectly support this mechanism. Paradoxically, plasma AGEs are lower in obese compared to lean subjects [49], probably since lipophilic AGEs are preferentially trapped into fat tissue [50]. Whether in the case of anorexia-associated low body fat content its trapping capacity is exceeded with a consequent increase in plasma AGEs concentrations remains to be elucidated in further studies. Nevertheless, an increase of plasma AGEs probably creates concentration gradients for diffusion of low molecular weight AGEs into saliva [25].

Total antioxidant capacity of plasma is a non-specific method assessing the overall endogenous and exogenous antioxidants [14, 15]. Glutathione is an important endogenous antioxidant protecting cells from oxidative damage [13]. Plasma concentrations of uric acid, bilirubin, and albumin - substances that serve as endogenous scavengers of ROS - were lower in the patients with AN than in the controls. Albeit their concentrations were within the age- and sex-specific reference ranges, their lower levels in girls with AN could contribute to diminished concentrations of markers of antioxidant status - TAC and GSH/GSSG ratio. Although we cannot fully explain higher urinary TAC in patients with AN, it might be caused by increased urinary excretion of these substances [51, 52]. On the contrary, salivary GSH/GSSG ratio in the patients with AN was elevated, which is a consequence of lower GSSG in these patients. Since, GSSG is generated during reduction of peroxides via glutathione peroxidise, lower GSSG could be associated with decreased glutathione peroxidise activity or its increased turnover that is in line with previous study [53]. It suggests that salivary antioxidants could mirror local status rather than systemic changes. Differences in the salivary antioxidants could be associated with alternations of periodontal status related to the changes in oral microbiome [54] that often occurs in patients with AN [35].

The advantage of this study is a reasonably large cohort of young girls with AN, and analysis of several markers of oxidative status in different body fluids. We provide the first data on carbonyl stress markers in AN. The main limitation of the study is its cross-sectional nature, allowing only for comments on associations. Although the power analysis calculated for unequal sizes of groups was sufficient, the size of control group is quite small.

Our present study is the first study assessing wide range markers of oxidative and carbonyl stress in different body fluids, which represents a basis for further research. It has a cross-sectional design, which does not allow to analyze the effect of weight gain on changes of oxidative stress markers. Therefore, our next study should have a longitudinal design with regular check-ups during the follow up. Moreover, the link between oxidative status, autophagy and an inflammation in AN should be elucidated.

## Conclusions

Our study demonstrated that anorexia-associated redox imbalance is accompanied by accumulation of oxidatively damaged proteins, which might exert a direct cytotoxic effect. Products of protein oxidation and glycation - AOPP, AGEs or fructosamines activate stress-sensitive pathways associated with increased expression of proinflammatory cytokines, growth factors, procoagulants, and adhesion molecules leading to micro- and macrovascular complications [55]. These pathways are also implicated in various chronic pathologies, such as inflammation, neurodegenerative, cardiovascular, nutritional diseases, atherosclerosis, etc. Thus, several complications of AN including dermatological signs, osteoporosis, myocardial atrophy, arrhythmias, nephropathy, gastrointestinal and neurological complications [2, 56] might be a direct consequence of accumulation of modified biomolecules over prolonged periods under diminished oxidative defence status. As disruption of redox homeostasis in young girls with AN appears to be an early finding, it could have a predictive value for development of disorders associated with AN. However, confirmation of this assumption requires longitudinal studies.

## Supplementary Information

**Additional file 1.**

**Additional file 2.**

## Data Availability

The datasets generated and/or analyzed during the current study are available from the corresponding author on reasonable request.

## Abbreviations

AGEs: Advanced glycation end-products
AGE-Fl: Advanced glycation end-products associated fluorescence
ALT: Alanine aminotransferase
AN: Anorexia nervosa
AOPP: Advanced oxidation protein products
AST: Aspartate aminotransferase
BMI: Body mass index
DSM-5: Diagnostic and Statistical Manual of Mental Disorders 5th Edition
eGFR: Estimated glomerular filtration rate
FRAP: Ferric reducing antioxidant power
GSH/GSSG: A ratio of reduced and oxidized glutathione
HDL-C: High density lipoprotein cholesterol
IQR: Interquartile range
MDA: Malondialdehyde
NICD: The National Institute of Children’s Diseases
PCR: A ratio of protein and creatinine
RNS: Reactive nitrogen species
ROS: Reactive oxygen species
SD: Standard deviation
TAC: Total antioxidant capacity
TAG: Triacylglycerols
TBARS: Thiobarbituric acid reactive substances
