# Supplementary material for: Oxidative status in plasma, urine and saliva of girls with anorexia nervosa and healthy controls: a cross-sectional study
Source: J Eat Disord. 2021 Apr 21;9:54. doi: 10.1186/s40337-021-00408-6 (PMC8059320; doi:10.1186/s40337-021-00408-6)
Supplement: Supplementary file 1 — Additional file 1. [file 40337_2021_408_MOESM1_ESM.docx]

Additional methods

As a marker of oxidative damage of lipids, TBARS was measured [9]. Twenty µl of samples and standards (1,1,3,3-tetraethoxypropane) were mixed with 30 µl of distilled water, 20 µl of 0.67% thiobarbituric acid and 20 µl of glacial acetic acid. After short mixing, the plates were incubated 95°C for 45 minutes. After that, 100 µl of n-butanol were added into all samples and standards and plates were centrifuged at 2000 g on 4°C for 10 minutes. Finally, 70 µl of upper phase were transferred into 96-well microstest plate and fluorescence was measured at _ex_ = 515 nm and λ_em_ = 535 nm.

As a marker of oxidative damage of proteins, AOPP was measured [10]. Two hundred µl of samples and standards (chloramine T mixed with 10 µl of potassium iodide) were mixed with 20 µl of glacial acetic acid. Absorbance was measured at 340 nm.

For AGE- Fl measurement, 20 µl of standards (modified bovine serum albumin) and samples were diluted in 180 µl of phosphate buffer saline in dark microtest plates (Sarstedt, Bratislava, Slovakia). After short vortexing, the fluorescence of Schiff base and Amadori products were measured at λ_ex_. = 370 nm and λ_em_. = 440 nm [11].

For fructosamine measurement 16 mmol/l 1-deoxy-morpholino-D-fructose was used as a standard. Twenty µl of samples and standards were mixed with 100 µl of 0.25 mM/l nitro blue tetrazolium. After incubation at 37°C for 15 minutes, absorbance was measured at 530 nm [12] .

As a marker of antioxidant status, FRAP was measured [14]. Briefly, 200 µl of fresh prepared and warmed (37°C) FRAP reagent was pipetted into microtest plate. Absorbance was measured at 593 nm and served as a blank. Afterwards, 20 µl of samples and standards (FeSO_4_*7H_2_O) were added. After short vortexing, absorbance was measured again at 593 nm. The initial absorbance that served as a blank was subtracted from the second absorbance.

For the measurement of TAC [15], 20 µl of samples and standards (trolox) were mixed with 200 µl of acetate buffer (pH=5.8). The absorbance was measured at 660 nm. Afterwards, 20 µl of 2,2'-azino-bis(3-ethylbenzthiazoline-6-sulphonic acid solution were added. The absorbance was measured again at 660 nm. In calculation, the initial absorbance that served as a blank was subtracted from the second absorbance.

For the measurement of GSH, 10 µl of samples were mixed with 10 µl of O-Phtalaldehyd solution (1 mg/ml) and 180 µl of the phosphate buffer solution (100 mM with 2.5 mM EDTA-Na_2_). After incubation at 37°C for 15 minutes, fluorescence was measured at ex.=350 nm, em.=460 nm. For the measurement of GSSG, 25 µl of samples were mixed with 10 µl of the N-ethylmaleimide (5 g/ml). After incubation for 40 minutes at room temperature, 10 µl of mixture were transferred into new microtest plate. Afterwards, 10 µl of the O-Phftalaldehyd (1 mg/ml) + 180 µl of NaOH (0.1 M) were added. The mixture was incubated for 15 minutes at the room temperature. Fluorescence was measured at ex.=350 nm, em.=460 nm. GSH/GSSG ratio was calculated [13].

To measure creatinine in urine samples, Jaffé method was used [29]. Briefly, 10 µl of samples and standards were mixed with 200 µl of fresh prepared working solution (NaOH and picric acid, 5:1 ratio). Absorbance was measured after 6 minutes at 492 nm.

To measure proteins in urine, 10 µl of samples and standards (bovine serum albumin) were mixed with 300 µl of pyrogallol red solution. After incubation at 37°C for 15 minutes, absorbance was measured at 595 nm [30].

Plasma proteins were measured using bicinchoninic acids assay. Briefly, 10 µl of samples and standards (bovine serum albumin) were mixed with 200 µl of fresh prepared working solution (bicinchoninic acid and copper sulphate, 49:1 ratio). The mixture was incubated for 30 minutes at 37°C. Absorbance was measured at 562 nm.
