# Supplementary material for: Oxidative status in plasma, urine and saliva of girls with anorexia nervosa and healthy controls: a cross-sectional study
Source: J Eat Disord. 2021 Apr 21;9:54. doi: 10.1186/s40337-021-00408-6 (PMC8059320; doi:10.1186/s40337-021-00408-6)
Supplement: Supplementary file 2 — Additional file 2. [file 40337_2021_408_MOESM2_ESM.docx]

Additional table: Regression analyses done between duration of AN and markers of oxidative stress in plasma, urine and saliva.

| Dependent variable | Beta | SE | 95% CI | p |
| --- | --- | --- | --- | --- |
| Plasma TBARS | 0.294 | 0.048 | 0.058 – 0.250 | ˂0.01 |
| Plasma AOPP | 0.264 | 0.008 | 0.007 – 0.038 | ˂0.01 |
| Plasma AGEs | 0.308 | 0.000 | 0.000 – 0.002 | ˂0.001 |
| Plasma FRUCTOS | 0.011 | 0.008 | -0.016 – 0.018 | ˃0.05 |
| Plasma FRAP | -0.169 | 1.125 | -4.206 – 0.257 | ˃0.05 |
| Plasma TAC | -0.250 | 3.449 | -11.850 – 1.979 | ˂0.001 |
| Plasma GSH/GSSG | -0.173 | 0.028 | -0.107 – 0.005 | ˃0.05 |
| Salivary TBARS | -0.016 | 0.006 | -0.012 – 0.010 | ˃0.05 |
| Salivary AOPP | -0.012 | 0.196 | -0.412 –0.367 | ˃0.05 |
| Salivary AGEs | -0.097 | 0.004 | -0.013 – 0.004 | ˃0.05 |
| Salivary fructos | -0.113 | 0.004 | -0.013 – 0.003 | ˃0.05 |
| Salivary FRAP | -0.091 | 1.499 | -4.323 – 1.625 | ˃0.05 |
| Salivary TAC | 0.178 | 2.548 | -0.475 – 9.639 | ˃0.05 |
| Salivary GSH/GSSG | 0.083 | 0.007 | -0.008 – 0.019 | ˃0.05 |
| Urinary TBARS | 0.114 | 0.046 | -0.043 – 0.140 | ˃0.05 |
| Urinary AOPP | 0.231 | 0.394 | 0.068 – 1.635 | ˂0.05 |
| Urinary fructos | -0.234 | 0.005 | -0.022 – -0.001 | ˂0.05 |
| Urinary FRAP | 0.161 | 26.217 | -13.109 – 91.181 | ˃0.05 |
| Urinary TAC | 0.253 | 19.798 | 8.096 – 86.838 | ˂0.05 |
| Urinary GSH/GSSG | -0.083 | 0.005 | -0.013 – 0.006 | ˃0.05 |

AGEs, advanced glycation end products; AN, anorexia nervosa; AOPP, advanced oxidation protein products; CI, confidence interval; FRAP, ferric reducing antioxidant power; FRUCTOS, fructosamines; GSH/GSSG, a ratio of reduced and oxidized glutathione; NS, non-significant; SE, standard error; TAC, total antioxidant capacity; TBARS, thiobarbituric acid reactive substance; duration of AN was considered as independent variable
